# Additives in foods marketed to children in Uruguay, an emerging Latin American country

**DOI:** 10.1017/jns.2024.98

**Published:** 2025-01-20

**Authors:** Florencia Alcaire, Ana Giménez, Gastón Ares

**Affiliations:** Sensometrics & Consumer Science, Instituto Polo Tecnológico de Pando, Facultad de Química, Universidad de la República, By Pass de Rutas 8 y 101 s/n, Pando CP 91000, Canelones, Uruguay

**Keywords:** Additives, Food environment, Food marketing, Labelling

## Abstract

Foods are frequently marketed to children through the inclusion of a wide range of elements on the packages. Several studies conducted in different countries around the globe have shown that products marketed to children are usually high in sugar and other nutrients associated with non-communicable diseases. The present exploratory cross-sectional study aimed at providing additional evidence on the composition of products marketed to children by analysing the disclosure of additives in such products. Data were collected at nine supermarkets in two Uruguayan cities between August and October 2021. All packaged products available in each supermarket were surveyed using a cell phone app, except for culinary ingredients. All the information from the labels was extracted. Products marketed to children were identified based on the presence of indicators of child-directed marketing on the package. The disclosure of additives was analysed based on the information available in the ingredient list. The prevalence of food additive disclosure was calculated. Of the 7,343 products included in the database, 573 (7.8%) were classified as marketed to children. Candies and cookies were the categories with the largest number of products marketed to children. The great majority of the products marketed to children disclosed at least one food additive (93.5%). These products tended to more frequently notify colourings, antioxidants, acidity regulators, raising agents, stabilisers, humectants, anticaking agents, and glazing agents compared to products not marketed to children. These findings underscore the need to expand the current scope of regulations on marketing targeted at children beyond nutrients to include additives potentially linked to adverse health effects, such as artificial colourings.

## Introduction

The diets of children worldwide largely deviate from international recommendations for healthy and sustainable diets^(1,2)^. Food systems are increasingly recognised as one of the major drivers of these deviations, as they are oriented towards the production of nutritionally unbalanced ultra-processed products^(3,4)^. The commercial practices of the food industry to develop, produce, and sell these products, including food marketing, introduce changes to the food environment and increase consumer demand^(5,6)^

Children are particularly vulnerable to the persuasive effects of food marketing^(7)^. Children’s exposure to food marketing is associated with increased preference, choice, consumption, and purchase requests^(8)^. Packaging has been identified as one of the key strategies of the food industry to market products to children^(9,10)^. Several studies conducted in different countries around the globe have shown that products marketed to children are usually unhealthy, as they are high in sugar and other nutrients associated with non-communicable diseases^(11–17)^. In addition, some studies have reported that products marketed to children tend to have a higher sugar content and a lower fibre content than those not marketed to this population group^(15,18–20)^.

Apart from their unfavourable nutritional composition, processed and ultra-processed products marketed to children may also contain food additives^(21)^, that is, substances intentionally added to foods for a technological purpose^(22)^. Concerns have been increasingly raised regarding the potential adverse health effects associated with food additives. Several functional classes of food additives have been associated with alterations in the intestinal microbiota, which can decrease the gut barrier function and activate inflammatory processes^(23,24)^. Through immune, endocrine, and neuronal pathways, these alterations could lead to an increased risk of obesity, type 2 diabetes, hypertension, Alzheimer’s disease, and other negative brain and behavioural consequences^(25,26)^. So far, dysbiosis has been reported as an effect of the consumption of sweeteners, emulsifiers, preservatives, colourings, and some other specific substances^(23,27,28)^. In addition, studies have reported associations between the consumption of sweeteners (aspartame, acesulfame-K, and sucralose), titanium dioxide, nitrites, and monosodium glutamate with an increased risk of various types of cancer^(29–31)^. In the specific case of children, the consumption of artificial colourings has been associated with neurobehavioral alterations: inattention, hyperactivity, and restlessness^(32)^.

The available evidence suggests that products marketed to children frequently contain food additives. A study conducted in California, USA, reported that 43.2% of the products targeted children contained artificial colours^(33)^. More recently, a study analysing the composition of biscuits commercialised in four European countries reported similarities in the most frequent additives included in products marketed to adults and children^(34)^. However, as far as it can be ascertained, only one study so far has performed a comprehensive analysis of the disclosure of food additives in products marketed to children. Kraemer et al. reported that the prevalence of additive disclosure in products marketed to infants and children accounted for 86%, being flavourings, emulsifiers, and colourings as the most frequent functional classes^(35)^. Considering a high prevalence of additives has been reported in processed and ultra-processed foods commercialised in Brazil, the United States, and France^(35–40)^, additional research is needed to obtain an in-depth understanding of the composition of foods marketed to children.

### Objectives and context

The present study aimed at assessing the disclosure of additives in packaged food products marketed to children in Uruguay, an emerging Latin American country. Specifically, the following objectives were sought (i) to characterise products marketed to children in the Uruguayan market, (ii) to estimate the prevalence of disclosure of additives in products marketed to children, (iii) to estimate the most frequently disclosed additives in products marketed to children, (iv) to compare the prevalence of additive disclosure between products marketed and not marketed to children.

Uruguay is a high-income country situated in the south-eastern region of South America. The country exhibits one of the highest prevalences of overweight and obesity across all age groups in the region: 16.2% among children aged 12–47 months, 39.4% among children aged 4–11 years, 33.6% among adolescents aged 13–17 years, and 65% among adults aged 19–65 years.^(41–43)^ Frequent consumption of ultra-processed products has been identified as a relevant behavioural risk factor contributing to these high rates of overweight and obesity^(44–46)^. To cope with this situation, Uruguay has implemented several policies aimed at fostering healthier food environments and reducing consumption of ultra-processed products. In 2014, the country implemented a policy to promote healthy eating habits in the school environment, which included the prohibition of the marketing of foods high in sugars, fats, and sodium in school environments.^(47)^ In 2018, Uruguay approved a front-of-package nutrition labelling policy that mandates the inclusion of warning labels on food products with excessive content of sugars, fat, saturated fat, and sodium, which entered into force in 2020.^(48)^ Additionally, from 2024, products featuring warning labels cannot be sold in primary and secondary schools.^(49)^ Despite these policy advancements, Uruguay has not implemented regulations or guidelines on food marketing, including restrictions on marketing targeted at children.

## Methods

The study relied on an exploratory cross-sectional design to assess the disclosure of food additives in processed and ultra-processed products marketed to children in Uruguay.

### Setting

Data were collected using a convenience sample of nine supermarkets, located in two cities in Uruguay: Montevideo (capital city) and Maldonado. The sample included stores of the six largest chains of large- and medium-sized supermarkets in the country, as well as stores of three additional chains of medium- and small-sized supermarkets. Data collection was conducted between August and October 2021.

### Data collection

Three data collectors surveyed all packaged products available in each supermarket using a cell phone app specially developed by the research team. Culinary ingredients (e.g. flour, oil, sugar, rice, unprocessed legumes, spices) were not included. Data collectors scanned the barcode of products and registered three pictures: front of the package, nutrient declaration, and ingredient list. These last pieces of information are compulsorily included in food packages in the country. The information was uploaded to an online database during data collection. Each product was registered the first time it was scanned.

After data collection was finalised, data from the pictures were extracted by three researchers. For each of the products registered in the database, the following information was manually extracted to an online spreadsheet: product name, company name, brand name, net weight, country of origin, ingredients, nutrition information (including portion size), description of label design, nutrition and health claims, any type of marketing claim, and presence of front-of-package warning labels for excess of sugar, fat, saturated fat, and/or sodium. Products were considered unique if they differed in at least one intrinsic characteristic (product name, company name, type of packaging, net weight, country of origin, nutrition information, ingredient list). For example, variations of products in package size or flavour were regarded as different, whereas products with different barcodes not differing in any characteristic were regarded as identical.

The quality of the database was checked by one of the researchers by sampling 5% of the database. Incongruencies between the pictures and the exported information were identified and corrected. The procedure was repeated until no differences were found.

### Data analysis

The key outcome of the present work was the percentage of products declaring food additives at the aggregate level and disaggregated by (sub)categories, type of additive, and whether products were marketed to children or not.

#### Identification of products marketed to children

**Figure 1.**
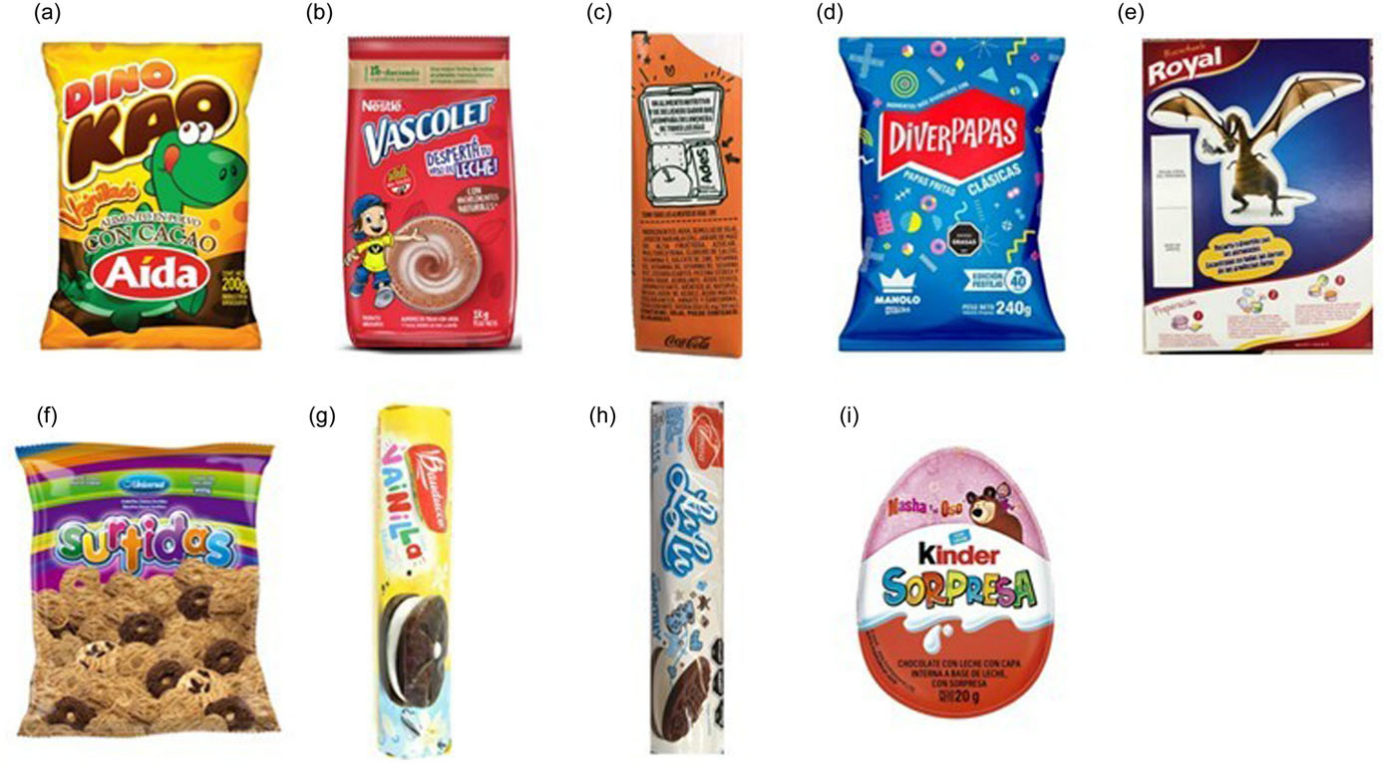
Examples of products including elements related to each of the indicators: (a) cartoon characters; (b) explicit references to childhood; (c) explicit references to school; (d) references to fun or games that appeal to children; (e) tie-ins with movies, toys, TV shows, or celebrities or that appeal to children; (f) Attractive or unconventional colours; (g) childish font; (h) other non-character-based graphic elements appealing to children; (i) gifts or toys.

Products marketed to children were identified based on the presence of indicators of child-directed marketing on the package. The following nine indicators were considered based on published research^(13,50,51)^: (i) cartoon characters; (ii) explicit references to childhood; (iii) explicit references to school; (iv) references to fun, games, or sports that appeal to children; (v) tie-ins with movies, toys, TV shows, sport personalities, or other celebrities that appeal to children; (vi) attractive or unconventional colours; (vii) childish font; (viii) other non-character-based graphic elements appealing to children (e.g. planes, rainbows, balloons, stars); (ix) gifts or toys. Examples of products including elements related to each of the indicators are shown in Fig. 1.

One of the researchers coded all the products based on the description of the label design and claims included in the spreadsheet and the pictures of the packages (in case of doubts). Binary variables were used to indicate whether a product included each of the indicators of child-directed marketing (0 = no, 1 = yes). The coding was revised by another researcher. Disagreements were found for 1% of the products and were solved by open-discussion until a consensus was reached. Products were regarded as marketed to children if they included at least one indicator of child-directed marketing.

#### Classification of products in categories and subcategories

Products were classified into categories and subcategories according to the guidance document describing the food categories in the European regulation of food additives^(52)^. One of the researchers used the product name and the pictures to classify each of the products. Minor changes to the original classification were made to fit the products available in the Uruguay market. The procedure described in Section 2.2.1 was used to check coding reliability. Disagreements on 3% of the products were solved by open discussion. The final categories are shown in Table 1.

**Table 1.** Number and percentage of products marketed to children per category and subcategory

Category/subcategory	Number of products	Products marketed to children	Percentage of all products marketed to children (%)	Percentage of products marketed to children within the (sub) category (%)
Confectionery	779	214	37.3	27.5
Chocolates	367	25	4.4	6.8
Candies	203	111	19.4	54.7
Cocoa and sweetened cocoa powder	66	37	6.5	56.1
Other confectionery items	61	8	1.4	13.1
Decorations, coatings, and fillings	53	24	4.2	45.3
Chewing gum	29	9	1.6	31.0
Bakery wares	1482	141	24.6	9.5
Cookies	484	105	18.3	21.7
Crackers or salty crackers’ sticks	328	0	0.0	0.0
Ready-to-eat sweet bakery wares	217	10	1.7	4.6
Bread	158	1	0.2	0.6
Alfajores	146	23	4.0	15.8
Readymade dough	71	0	0.0	0.0
Powdered mixes to prepare sweet bakery wares	45	2	0.3	4.4
Ready-to-eat salty bakery wares	18	0	0.0	0.0
Powdered mixes to prepare salty bakery wares	15	0	0.0	0.0
Cereals and cereal products	560	57	9.9	10.2
Pasta	208	0	0.0	0.0
Cereal bars	146	8	1.4	5.5
Breakfast cereals	138	49	8.6	35.5
Mixes to prepared cereal dishes	37	0	0.0	0.0
Whole, broken, or flaked grain	13	0	0.0	0.0
Mixes of flour and other milled products and starches	9	0	0.0	0.0
Powdered mixes to prepare pasta	5	0	0.0	0.0
Other cereal products	4	0	0.0	0.0
Dairy products and analogues	710	55	9.6	7.7
Cheese and cheese products	327	1	0.2	0.3
Yogurt	155	18	3.1	11.6
Dairy analogues	81	12	2.1	14.8
Sweetened condensed milk	65	2	0.3	3.1
Dairy desserts	55	17	3.0	30.9
Flavoured milk	13	5	0.9	38.5
Other dairy products	13	0	0.0	0.0
Cream and cream powder	1	0	0.0	0.0
Ready-to-eat savouries and snacks	415	30	5.2	7.2
Vegetable, cereal, flour, or starch-based snacks	251	30	5.2	12.0
Processed nuts	164	0	0.0	0.0
Desserts	227	26	4.5	11.5
Powder mixes to prepare desserts	153	23	4.0	15.0
Ready-to-eat desserts	74	3	0.5	4.1
Beverages	525	25	4.4	4.8
Fruit and vegetable juice or nectars and similar products	211	9	1.6	4.3
Soft drinks	166	10	1.7	6.0
Powdered Juices	73	6	1.0	8.2
Coffee, coffee extracts, coffee extracts, coffee substitutes, coffee mixes, and mixes for hot beverages	36	0	0.0	0.0
Other beverages	25	0	0.0	0.0
Sports drinks	10	0	0.0	0.0
Energy drinks	4	0	0.0	0.0
Ice cream and popsicles	230	10	1.7	4.3
Ice cream	206	2	0.3	1.0
Water ice cream	24	8	1.4	33.3
Meat and analogues	299	5	0.9	1.7
Sausages, cooked and cured meat products	125	0	0.0	0.0
Cooked, heat-treated, and canned meat	57	0	0.0	0.0
Hamburgers	46	1	0.2	2.2
Meat analogues	41	0	0.0	0.0
Other meat products	29	4	0.7	13.8
Unprocessed meat	1	0	0.0	0.0
Foods for infants and young children	10	4	0.7	40.0
Puree	9	3	0.5	33.3
Growing-up milk	1	1	0.2	100.0
Sauces	456	3	0.5	0.7
Sauces ready-to-eat	435	3	0.5	0.7
Dehydrated or powdered mixes to prepared sauces	21	0	0.0	0.0
Fruit, vegetables, legumes, nuts, and seeds	1054	2	0.3	0.2
Fruit and vegetables in vinegar, oil, or brine	458	0	0.0	0.0
Jam, jellies, marmalades, and similar products	209	0	0.0	0.0
Processed potato products	99	2	0.3	2.0
Fruit and vegetables in syrup or sweet liquid	91	0	0.0	0.0
Processed tomato products	90	0	0.0	0.0
Unprocessed nuts and seeds	39	0	0.0	0.0
Frozen fruits and vegetables	23	0	0.0	0.0
Other fruit and vegetable preparations (e.g. eggplant or olive paste)	16	0	0.0	0.0
Processed vegetable products	15	0	0.0	0.0
Nut and seeds butters or spreads	13	0	0.0	0.0
Dried fruit and vegetables	1	0	0.0	0.0
Fish and fisheries products	279	1	0.2	0.4
Processed fish and fishery products including molluscs and crustaceans	246	0	0.0	0.0
Other fish or molluscs products	32	1	0.2	3.1
Unprocessed fish and fisheries products	1	0	0.0	0.0
Other processed foods	152	0	0.0	0.0
Frozen and ready-to-eat meals (e.g. frozen pizza, ready-to-eat sandwich)	152	0	0.0	0.0
Soups and broths	109	0	0.0	0.0
Soups and broths	109	0	0.0	0.0
Seasonings and condiments	27	0	0.0	0.0
Seasonings and condiments	27	0	0.0	0.0
Fats and oils and fat and oil emulsions	24	0	0.0	0.0
Butter and concentrated butter and butter oil and anhydrous milk fat	12	0	0.0	0.0
Other fat and oil emulsion	12	0	0.0	0.0
Sugars, syrups, honey	5	0	0.0	0.0
Sugars, syrups, honey	5	0	0.0	0.0
Grand total	7343	573	100.0	7.8

#### Identification of food additives

A computer-assisted identification of additives was performed using the packages stringr and tokenizers in R language^(53)^. First, a list of all the words included in the ingredient list was obtained. Spell check and correction of the database were performed by one of the researchers. Then, a list of all the ingredients included in the database was obtained by identifying all the text strings separated by commas in the ingredient list. The list was manually inspected by two of the researchers and regular expressions corresponding to all the additives included in the Food Additive Index of Codex Alimentarius, which matched the Uruguayan legislation^(54)^ were manually identified. Then, searches for each of the expressions were performed and binary variables were used to code whether each product notified each of the additives.

Additives were then grouped into fifteen functional classes: acidity regulators, anticaking agents, antioxidants, colourings, enzymes, flavourings, flavour enhancers, glazing agents, humectants, preservatives, propellants, raising agents, stabilisers/emulsifiers/thickeners/gelling or firming agents, sweeteners, and others. When an additive could be classified into more than one functional class, it was included in the class most frequently declared in the products that notified that specific additive. Binary variables were created to indicate whether each of the products notified an additive included in each functional class or not (1/0).

#### Descriptive statistics

Descriptive statistics were used to summarise the data. Absolute and relative frequencies were calculated for binary variables and averages and standard deviations were calculated for continuous variables.

#### Inferential statistics for comparing products marketed and not marketed to children

Fisher’s exact test was used to compare the disclosure of additives in products marketed and not marketed to children. A 5% significance level was considered. Comparisons were made at the aggregate level and for specific subcategories with at least twenty products marketed to children.

## Results

A total of 7,343 unique products were surveyed across eighteen categories and seventy-four product subcategories (Table 1). Of the 7,343 products, 573 (7.8%) displayed at least one indicator of child-directed marketing and, therefore, were classified as marketed to children.

Products identified as marketed to children were found in thirteen of the eighteen categories (Table 1). The majority of the products marketed to children corresponded to candies (19.4%) and cookies (18.3%). As shown in Table 1, ten subcategories had more than 30% of the products classified as marketed to children: candies; cocoa and sweetened cocoa powder; decorations, coatings and fillings; chewing gum; breakfast cereals; dairy desserts; flavoured milk; water ice cream; puree for infants and young children; growing-up milk.

Regarding the country of origin of the products, 44.5% had been packaged in Uruguay, 25.1% in Brazil, 22.0% in Argentina, and 8.4% in other countries (Spain, Colombia, Serbia, Denmark, Chile, Mexico, Germany, USA, and Turkey).

### Prevalence of indicators of child-directed marketing on the packages

Products marketed to children included an average of 2.1 indicators of child-directed marketing (SD = 0.8) on the package. Two was the most frequent number of indicators (41.9%), whereas 28.8% of the products included three indicators, 25.5% included only one, and 3.8% included four.

The most frequent indicator of child-directed marketing included on the packages was childish font (76.6%), followed by attractive colours (58.1%) and cartoon characters (41.2%) (Table 2). On the contrary, references to fun, tie-ins, and references to school were only found in less than 10% of the products. As shown in Table 2, there was variation in the prevalence of indicators of child-directed marketing across categories. For example, references to childhood tended to be more frequent in foods for infants and young children and sauces, references to fun in ready-to-eat savouries and snacks, tie-ins in desserts, and references to school in dairy products and analogues.

**Table 2.** Prevalence of indicators of child-directed marketing on the packages of products identified as marketed to children (n = 573), expressed as percentage of products, at the aggregate level and separately for each category

Category	Number of products	Childish font	Attractive colours	Cartoon characters	Other graphic elements appealing to children	References to childhood	References to fun or games	Gifts, toys or games	Tie-ins	References to school
Confectionery	214	87.4	81.3	42.5	8.4	10.7	1.4	1.4	0.5	0.0
Bakery wares	141	73.0	38.3	24.1	12.8	11.3	8.5	1.4	1.4	0.0
Cereals and cereal products	57	71.9	50.9	66.7	21.1	7.0	1.8	0.0	0.0	0.0
Dairy products and analogues	55	69.1	52.7	67.3	20.0	9.1	3.6	0.0	18. 2	12.7
Ready-to-eat savouries and snacks	30	66.7	60.0	30.0	20.0	6.7	23.3	0.0	0.0	0.0
Desserts	26	61.5	15.4	38.5	19.2	7.7	0.0	42.3	0.	0.0
Beverages	25	52.0	64.0	44.0	16.0	8.0	0.0	0.0	0.0	0.0
Ice cream and popsicles	10	80.0	60.0	0.0	20.0	0.0	0.0	0.0	0.0	0.0
Meat and analogues	5	100.0	20.0	60.0	20.0	20.0	0.0	0.0	0.0	0.0
Foods for infants and young children	4	75.0	50.0	0.0	25.0	75.0	0.0	0.0	0.0	0.0
Sauces	3	100.0	0.0	0.0	0.0	100.0	0.0	0.0	0.0	0.0
Fruit, vegetables, legumes, nuts and seeds	2	50.0	0.0	100.0	0.0	0.0	0.0	0.0	0.0	0.0
Fish and fisheries products	1	100.0	0.0	100.0	100.0	0.0	0.0	0.0	0.0	0.0
Grand total	573	76.6	58.1	41.2	13.8	10.6	4.4	2.8	2.3	1.2

### Disclosure of additives

The great majority of the products marketed to children (93.5%) notified at least one additive. A wide range of additives were identified (Table 3). The ten most frequently notified were citric acid (INS330, 34.2%), lecithin (INS322, 32.3%), sodium carbonates (INS500, 25.8%), tartrazine (INS102, 22.5%), gelatine (INS428, 21.8%), Allura red AC (INS129, 21.1%), Brilliant blue FCF (INS133, 21.1%), ammonium carbonates (INS503, 18.3%), Sunset yellow (INS110, 15.7%), and enzymatically modified starch (INS1405, 12.4%). When additives were grouped according to their function, it was found that more than half of the products marketed to children contained at least one flavouring (86.2%), stabiliser/emulsifier/thickeners/gelling or firming agents (74.9%), and/or colouring (52.5%).

**Table 3.** Percentage of products targeted and not marketed to children notifying different food additives

Functional class	Food additive	Marketed to children (n = 573)	Not marketed to children (n = 6770)
Flavourings*		86.2	46.6
Stabilisers/emulsifiers/thickeners/gelling or firming agents*		74.9	54.1
	INS322 Lecithin*	32.3	19.2
	INS428 Gelatine*	21.8	3.4
	INS1405 Enzyme treated starch*	12.4	6.5
	INS414 Acacia gum*	11.2	2.1
	INS476 Polyglycerol esters of interesterified ricinoleic acid	6.6	4.9
	INS471 Mono- and di-glycerides of fatty acids*	6.5	9.6
	INS407 Carrageenan	5.9	4.5
	INS440 Pectins*	5.1	3.3
	INS412 Guar gum	4.9	6.7
	INS415 Xanthan gum*	4.7	8.8
	INS420 Sorbitol	3.7	3.8
	INS466 Carboxymethylcellulose*	3.1	5.1
	INS339 Sodium phosphates	3.0	2.0
	INS481 Sodium lactylate	2.3	1.4
	INS451 Triphosphates	1.9	1.9
	INS472 Acetic/lactic/citric/tartaric and fatty acid esters of glycerol	1.9	3.2
	INS492 Sorbitan tristearate*	1.4	0.3
	INS475 Polyglycerol esters of fatty acids	1.2	0.5
	INS340 Potassium phosphates	1.0	0.8
	INS509 Calcium chloride*	1.0	4.0
	INS406 Agar	0.9	1.1
	INS418 Gellan gum	0.9	0.5
	INS1200 Polydextrose*	0.9	3.0
	INS508 Potassium chloride	0.7	1.5
	INS515 Potassium sulphate*	0.5	0.0
	INS541 Sodium aluminium phosphate	0.5	0.5
	INS433 Polysorbate 80*	0.3	1.4
	INS452 Polyphosphates*	0.3	3.9
	INS477 Propylene glycol esters of fatty acids	0.3	0.6
	INS410 Locust bean gum	0.2	0.7
	INS442 Ammonium salts of phosphatidic acid	0.2	0.0
	INS464 Hydroxypropyl methylcellulose	0.2	0.6
	INS470 Salts of fatty acids	0.2	0.3
	INS491 Sorbitan monostearate	0.2	0.2
	INS390 Dioctadecyl thiodipropionate	0.0	0.0
	INS399 Calcium lactobionate	0.0	0.0
	INS400 Alginic acid	0.0	0.0
	INS401 Sodium alginate*	0.0	0.8
	INS404 Calcium alginate	0.0	0.0
	INS405 Propylene glycol alginate	0.0	0.0
	INS413 Tragacanth gum	0.0	0.0
	INS416 Karaya gum	0.0	0.0
	INS417 Tara gum	0.0	0.3
	INS435 Polysorbate 60	0.0	0.1
	INS444 Sucrose acetate isobutyrate	0.0	0.0
	INS445 Glycerol esters of wood rosins	0.0	0.0
	INS461 Methylcellulose	0.0	0.2
	INS463 Hydroxypropyl cellulose	0.0	0.3
	INS482 Calcium lactylate	0.0	0.3
	INS494 Sorbitan monooleate	0.0	0.0
	INS504 Magnesium carbonate	0.0	0.2
	INS516 Calcium sulphate	0.0	0.2
	INS517 Ammonium sulphate	0.0	0.0
	INS542 Bone phosphate	0.0	0.0
	INS543 Sorbitan monooleate	0.0	0.1
	INS576 Sodium gluconate	0.0	0.1
	INS577 Potassium gluconate	0.0	0.1
	INS579 Ferrous gluconate	0.0	0.1
	INS900 Polydimethylsiloxane	0.0	0.3
	INS1414 Acetylated distarch phosphate	0.0	0.0
	INS1518 Triacetin	0.0	0.0
Colourings*		52.5	21.5
	INS102 Tartrazine*	22.5	5.0
	INS129 Allura red AC*	21.1	1.3
	INS133 Brilliant blue FCF*	21.1	1.7
	INS110 Sunset yellow*	15.7	3.5
	INS171 Titanium dioxide*	10.3	1.4
	INS150 Caramel*	9.1	5.2
	INS120 Carmines*	8.4	3.5
	INS160 Carotenes	5.9	6.0
	INS100 Curcumin*	5.4	2.1
	INS124 Ponceau 4R*	4.9	2.3
	INS132 Indigotine*	4.2	0.5
	INS127 Erythrosine*	3.3	0.2
	INS141 Chlorophyll copper complex*	1.7	0.1
	INS122 Azorubine*	1.7	0.2
	INS123 Amaranth	1.6	1.1
	INS163 Anthocyanins*	0.5	0.1
	INS174 Silver*	0.3	0.0
	INS153 Carbon black	0.3	0.0
	INS125 Ponceau SX	0.2	0.0
	INS104 Quinoline red	0.0	0.0
	INS130 Alizarin red	0.0	0.0
	INS161 Flavoxanthin	0.0	0.0
	INS180 Lithol rubine BK	0.0	0.0
	INS162 Beet red	0.0	0.1
	INS101 Riboflavin	0.0	0.1
	INS140 Chlorophyll	0.0	0.1
Antioxidants*		41.0	31.3
	INS330 Citric acid*	34.2	23.3
	INS321 BHT*	4.2	1.8
	INS320 BHA	2.4	1.5
	INS300 Ascorbic acid*	2.1	7.6
	INS306 Alpha-tocopherol	1.9	1.5
	INS385 Calcium disodium EDTA	1.9	1.7
	INS304 Ascorbyl palmitate*	1.7	0.5
	INS316 Sodium erythorbate	1.0	1.5
	INS319 TBHQ	1.0	0.5
	INS310 Propyl gallate	0.5	0.3
	INS301 Sodium ascorbate	0.3	0.3
	INS307 Tocopherols, mixed	0.3	0.2
	INS303 Potassium ascorbate	0.0	0.0
	INS386 Disodium EDTA*	0.0	0.8
Raising agents*		27.6	17.5
	INS500 Sodium carbonates*	25.8	14.3
	INS503 Ammonium carbonates*	18.3	6.7
	INS450 Diphosphates	7.9	6.0
	INS501 Potassium carbonates	0.2	0.4
	INS920 Cysteine	0.0	0.1
Acidity regulators*		20.2	11.3
	INS270 Lactic acid*	8.4	3.9
	INS331 Sodium citrate*	8.2	4.4
	INS296 Malic acid*	6.5	0.9
	INS325 Sodium lactate*	4.5	0.6
	INS297 Fumaric acid*	3.8	0.6
	INS524 Sodium hydroxide*	0.7	0.1
	INS338 Phosphoric acid	0.5	1.1
	INS575 Glucono-delta lactone	0.5	0.3
	INS327 Calcium lactate	0.2	0.1
	INS332 Potassium citrate	0.2	0.0
	INS333 Calcium citrate	0.2	0.0
	INS260 Acetic acid, glacial*	0.0	0.3
	INS262 Sodium acetate	0.0	0.1
	INS334 Tartaric acid	0.0	0.2
	INS336 Potassium tartrate	0.0	0.1
	INS526 Calcium hydroxide	0.0	0.0
	INS528 Magnesium hydroxide	0.0	0.0
Anticaking agents*		18.0	9.4
	INS341 Calcium phosphates*	8.7	5.9
	INS170 Calcium carbonates*	7.2	1.3
	INS553 Magnesium silicate*	2.8	0.0
	INS551 Silicon dioxide	1.2	2.1
	INS460 Cellulose	0.5	0.4
	INS552 Calcium silicate	0.0	0.0
Preservatives*		13.8	29.8
	INS202 Potassium sorbate*	8.9	17.6
	INS282 Calcium propionate*	3.8	7.3
	INS211 Sodium benzoate*	1.7	4.7
	INS200 Sorbic acid	4.7	4.2
	INS250 Sodium nitrite*	0.0	2.4
	INS223 Sodium metabisulphite	0.9	2.2
	INS251 Sodium nitrite*	0.0	1.1
	INS281 Sodium propionate	1.2	0.6
	INS222 Sodium bisulphite	0.0	0.4
	INS224 Potassium metabisulphite	0.0	0.3
	INS234 Nisin*	0.0	0.3
	INS220 Sulphur dioxide	0.0	0.2
	INS252 Potassium nitrate	0.0	0.1
	INS235 Natamycin	0.0	0.1
	INS212 Potassium benzoate	0.0	0.1
	INS201 Sodium sorbate	0.0	0.0
	INS210 Benzoic acid	0.0	0.0
	INS214 Ethyl para-hydroxybenzoate	0.0	0.0
Glazing agents*		11.0	0.4
	INS903 Carnauba wax*	9.8	0.3
	INS904 Shellac*	3.0	0.2
	INS901 Beeswax*	2.6	0.0
	INS905 Mineral oil*	0.3	0.0
Humectants*		6.6	2.6
	INS422 Glycerine*	6.6	2.2
	INS1520 Propylene glycol	1.2	0.5
Sweeteners*		6.5	9.7
	INS950 Acesulphame potassium*	2.6	5.1
	INS951 Aspartame	2.3	2.5
	INS955 Sucralose*	1.9	5.9
	INS952 Cyclamate	1.0	0.4
	INS954 Saccharin	1.0	0.5
	INS960 Steviol glycosides	0.9	1.3
	INS965 Maltitol	0.9	1.8
	INS421 Mannitol	0.9	1.8
	INS967 Xylitol	0.5	0.3
	INS953 Isomalt	0.0	0.1
	INS966 Lactitol	0.0	0.1
	INS968 Erythritol	0.0	0.4
Flavour enhancers*		3.5	6.3
	INS621 Monosodium L-glutamate*	3.5	6.1
	INS627 Disodium 5’-guanylate	1.0	1.3
	INS631 Disodium 5’-inosinate	1.0	2.3
	INS624 Monoammonium L-glutamate*	0.5	0.0
	INS620 L-glutamic acid*	0.3	0.0
	INS635 Disodium 5’-ribonucleotides	0.2	0.1
	INS626 Guanylic acid	0.0	0.0
	INS630 Inosinic acid	0.0	0.0
Enzymes*		0.3	2.0
	INS1101 Protease	0.3	0.7
	INS1100 Amylase*	0.0	1.1
	INS1103 Invertase	0.0	0.2
	INS1104 Lipase	0.0	0.1
	INS1105 Lisozyme	0.0	0.1
Propellants*		0.0	0.7
	INS290 Carbon dioxide*	0.0	0.6
	INS942 Nitrous oxide	0.0	0.0
Others		0.0	0.2
	INS927 Azodicarbonamide	0.0	0.2
Any food additive*		93.5	80.2

*Note*: Additives or additive classes highlighted with * statistically significantly differ in their frequency of disclosure between products targeted and not marketed to children according to Fisher’s exact test at 0.05.

**Figure 2.**
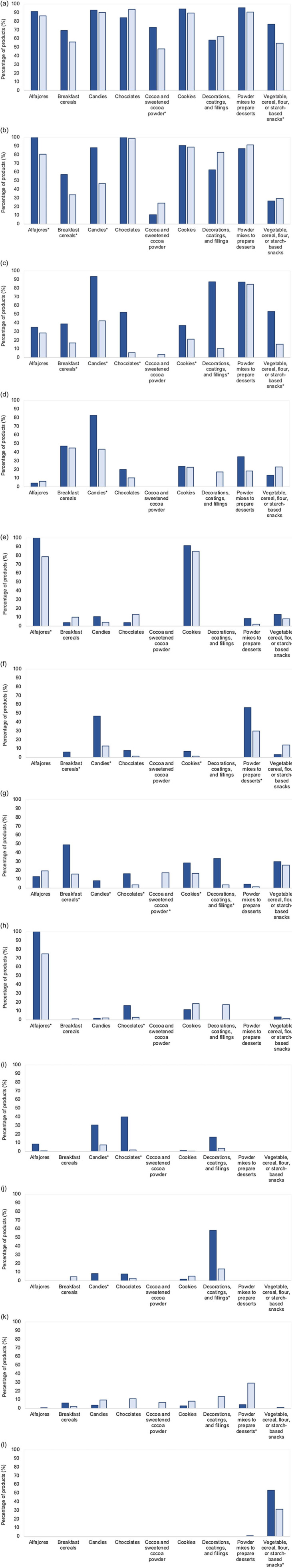
Percentage of products marketed (dark blue) and not marketed (light blue) to children notifying different classes of food additives: (a) flavourings, (b) stabilisers/emulsifiers/thickeners/gelling or firming agents, (c) colourings, (d) antioxidants, (e) raising agents, (f) acidity regulators, (g) anticaking agents, (h) preservatives, (i) glazing agents, (j) humectants, (k) sweeteners, (l) flavour enhancers. *Note:* Product subcategories highlighted with * statistically significantly differ in the frequency of notification of the class of food additives according to Fisher’s exact test at 0.05. The specific additives included within each functional class are shown in Table 3.

Compared to products not marketed to children, those that included indicators of child-directed marketing on the package statistically significantly (p < 0.001) notified additives more frequently in general. The same result was found for specific functional classes: colourings, antioxidants, acidity regulators, raising agents, stabilisers, humectants, anticaking agents, glazing agents. The opposite difference was found for preservatives, flavour enhancers, sweeteners, enzymes, and propellants. When the comparisons were performed at the level of specific subcategories, all classes of additives tended to be more frequently notified for products marketed to children (Fig. 2). The only exception to this trend were sweeteners, which were more frequently notified in products not marketed to children for powder mixes to prepare desserts.

## Discussion

The present study contributes to the literature by analysing the disclosure of food additives in products marketed to children commercialised in the Uruguayan market. The percentage of products marketed to children accounted for 7.8% of the database, which is in the middle of the range of values reported by recent studies conducted in Slovenia, Brazil, Australia, and Spain: from 4.1% to 17.5%^(11,13,15,17)^. The strategies used to target products to children were mainly based on graphic design and the inclusion of cartoon characters, in line with the existing evidence^(9,14,50,55–57)^.

Most of the products marketed to children corresponded to discretionary foods, such as candies, cookies, breakfast cereals, savoury snacks, desserts, and chocolates. These categories have been identified as those that have the largest contribution to the food industry’s expenditure on marketing marketed to children and adolescents^(58)^. The majority of the products marketed to children are consumed as snacks, which may reinforce children’s current tendency to frequently snack on processed and ultra-processed products with excessive content of nutrients associated with non-communicable diseases^(59–62)^. The similarities in the type of products marketed to children suggest that the food industry engages in consistent marketing strategies across the globe, regardless of the countries’ traditions, regulations, and market size.

The present study makes a relevant contribution to the literature by performing a comprehensive analysis of the disclosure of additives in products marketed to children. Results showed that 93.5% of products marketed to children contained at least one food additive. This percentage is slightly higher than that reported by Kraemer et al. in a recent study analysing the prevalence of food additives in products marketed to infants and children in the Brazilian market (86%).^(35)^ The prevalence of additive disclosure reported in the present work is also similar to that reported by other authors when analysing ultra-processed products^(40,63)^. Other authors have reported lower prevalences when analysing a wider range of products, including culinary ingredients and minimally processed products^(38–40)^.

Results from the present work showed that products marketed to children were more likely to contain food additives compared to the rest of the products commercialised in the marketplace. This is concerning given the emerging evidence on the potential medium- and long-term negative health consequences of some food additives^(23–28)^. In particular, products marketed to children were more likely to notify food additives than similar non-child products within the same subcategory. This suggests that the use of additives in products marketed to children may not be always fully justified, as required by the General standard for food additives CODEX STAN 192-1995^(22)^. In this sense, flavourings and colourings were two of the most frequently notified functional classes in products marketed to children. These functional classes of additives are not necessary from a technological point of view and are only added to add flavours and colours not conveyed by the ingredients used in product manufacture.

The great majority of the products marketed to children (86.2%) notified the addition of flavourings in the ingredient list. The percentage of products notifying this functional class of additives was higher among products marketed to children compared to non-child products, as well as higher than the prevalence reported for products commercialised in the USA^(36)^. Although these additives have not been associated with negative health consequences yet, they may interfere with the gut-brain signalling of food reward^(64,65)^. A better understanding of the effect of flavourings on the gut-brain pathway is critical to evaluate their safety, particularly during childhood. In addition, exposure to artificial flavourings from early childhood may be detrimental for the development of a preference for the flavour of natural foods, such as fruits and vegetables^(66,67)^.

Artificial colourings, such as Tartrazine, Allura red, Brilliant blue, and Sunset yellow were the most frequently notified type of colourings in products marketed to children. A high prevalence of these additives was previously reported in products marketed to children in the USA^(33)^. Artificial colourings lack nutritional and health benefits and have been associated with neurobehavioral alternations in children^(21,32,68,69)^. Titanium dioxide is another food colouring of concern, as it has been increasingly associated with disorders of the intestinal barrier and colorectal cancer^(70,71)^, and is no longer considered safe by the European Food and Safety Authority^(72)^. This additive was notified in 10.3% of the products marketed to children and only 1.4% of the products not marketed to this vulnerable population.

Emerging evidence also suggests that chronic consumption of some emulsifiers and preservatives is linked to negative health consequences through immune, endocrine, and neuronal pathways^(23–26)^. Results from the present work showed that several additives within these functional classes are used in more than 5% of the products marketed to children (e.g. polyglycerol esters of interesterified ricinoleic acid, mono- and di-glycerides of fatty acids, carrageenan, potassium sorbate), This suggests that stricter regulations on the use of additives in products marketed to children may be needed.

Results from the present work suggest that consumption of products marketed to children may imply exposure to a great diversity of food additives, which could have synergistic effects^(73)^. This exposure deserves special in the case of children because of their lower body weight and longer lifetime exposure compared to adults^(21,69)^. Marketing foods with unnecessary food additives (e.g. food colourings) to appeal children raise ethical issues. Manufacturing and marketing products with potential negative consequences for children’s’ health can be regarded as a breach to the convention of the rights of the child, which require that children’s best interests are taken into account in all actions relevant to them^(74)^. According to Article 24, governments should implement actions to address the underlying determinants of health and enable children to achieve the highest standard of health^(74)^. Therefore, regulatory strategies are needed to reduce the availability of products containing food additives with potential negative health consequences to children. Argentina, Chile, and Mexico have implemented packaging regulations that limit the use of child-directed marketing strategies, such as cartoon characters, child figures, tie-ins, toys, and games to promote energy dense products high in sugars, sodium, and fat and sugar across all media, including packaging^(75–77)^. The implementation of this policy in Chile led to a reduction in the percentage of cereals high in sugar featuring child-directed marketing strategies from 43% to 15%^(78)^. The findings of this study highlight the necessity of broadening the scope of restrictions on child-targeted marketing to include not only nutrient content but also additives associated with potential adverse health effects, such as artificial colourings.

Despite its relevance and novelty, the present study has a series of limitations. Although data collection was performed at nine supermarkets with different characteristics, the database does not include all the products commercialised in Uruguay. Additionally, the study only focuses on the disclosure of additives on food labels and does not include any chemical analyses to identify them in the analysed products. For this reason, the concentration of additives was not considered, as this information is not included on product labels.

## Conclusions

The present study provided a comprehensive overview of the availability of products marketed to children in the Uruguayan market. Results extend the existing literature on the composition of such products by showing that the presence of indicators of child-directed marketing is associated with a frequent disclosure of food additives, particularly flavouring, stabilisers/emulsifiers/thickeners/gelling or firming agents, colourings, and antioxidants. Considering the growing evidence on the potential negative health effects of some food additives, these results raise ethical concerns over the practices of the food industry and stress the need to develop comprehensive packaging regulations to protect children’s health. Results from the present underscore the need for additional research on the potential risks associated with the consumption of additives in children.
